# Neuropsychological test validation of speech markers of cognitive impairment in the Framingham Cognitive Aging Cohort

**DOI:** 10.37349/emed.2021.00044

**Published:** 2021-06-30

**Authors:** Larry Zhang, Anthony Ngo, Jason A. Thomas, Hannah A. Burkhardt, Carolyn M. Parsey, Rhoda Au, Reza Hosseini Ghomi

**Affiliations:** 1 Department of Intelligent Systems Engineering, Indiana University Bloomington, Bloomington, Indiana 47408, United States; 2 Department of Informatics, Indiana University Bloomington, Bloomington, Indiana 47408, United States; 3 Department of Statistics, University of Washington, Seattle, Washington 98195-0005, United States; 4 Department of Biomedical Informatics and Medical Education, University of Washington Seattle Campus, Seattle, Washington 98195-0005, United States; 5 Department of Neurology, University of Washington, Seattle, Washington 98195-0005, United States; 6 Department of Anatomy and Neurobiology, Neurology, and Epidemiology, Boston University Schools of Medicine and Public Health, Boston, Massachusetts 02118, United States

**Keywords:** Digital biomarkers, cognitive impairment, dementia, automated speech analysis, neurocognitive testing

## Abstract

**Aim:**

Although clinicians primarily diagnose dementia based on a combination of metrics such as medical history and formal neuropsychological tests, recent work using linguistic analysis of narrative speech to identify dementia has shown promising results. We aim to build upon research by Thomas JA & Burkardt HA et al. (J Alzheimers Dis. 2020;76:905-22) and Alhanai et al. (arXiv:1710.07551v1. 2020) on the Framingham Heart Study (FHS) Cognitive Aging Cohort by 1) demonstrating the predictive capability of linguistic analysis in differentiating cognitively normal from cognitively impaired participants and 2) comparing the performance of the original linguistic features with the performance of expanded features.

**Methods:**

Data were derived from a subset of the FHS Cognitive Aging Cohort. We analyzed a sub-selection of 98 participants, which provided 127 unique audio files and clinical observations (*n* = 127, female = 47%, cognitively impaired = 43%). We built on previous work which extracted original linguistic features from transcribed audio files by extracting expanded features. We used both feature sets to train logistic regression classifiers to distinguish cognitively normal from cognitively impaired participants and compared the predictive power of the original and expanded linguistic feature sets, and participants’ Mini-Mental State Examination (MMSE) scores.

**Results:**

Based on the area under the receiver-operator characteristic curve (AUC) of the models, both the original (AUC = 0.882) and expanded (AUC = 0.883) feature sets outperformed MMSE (AUC = 0.870) in classifying cognitively impaired and cognitively normal participants. Although the original and expanded feature sets had similar AUC, the expanded feature set showed better positive and negative predictive value [expanded: positive predictive value (PPV) = 0.738, negative predictive value (NPV) = 0.889; original: PPV = 0.701, NPV = 0.869].

**Conclusions:**

Linguistic analysis has been shown to be a potentially powerful tool for clinical use in classifying cognitive impairment. This study expands the work of several others, but further studies into the plausibility of speech analysis in clinical use are vital to ensure the validity of speech analysis for clinical classification of cognitive impairment.

## Introduction

Dementia is a syndrome that involves loss of cognitive function including memory, comprehension, and orientation, to a degree that impacts independent functioning. Dementia poses a significant burden both socially and economically. According to the World Health Organization, dementia affects around 50 million people worldwide, with nearly 10 million new cases per year [1]. The most common cause of dementia is Alzheimer’s disease (AD). AD is the sixth leading cause of death in the United States and the fifth leading cause of death among Americans age 65 and older [2]. The burden of dementia reaches further than just diagnosed patients. An estimated 16 million caregivers, often family members and friends, provide informal unpaid care for people with dementia, providing an estimated 18.6 billion hours of care valued at nearly $244 billion [3]. Early and accurate detection of cognitive decline can help promote optimal management of the disease, reducing the risk of accidents and injuries, as well as improving the experience of families and caregivers [4]. Thus, there is a need for simple, accessible, and noninvasive methods to determine cognitive status and the risk of developing dementia [5].

There is no single test for dementia. Clinicians diagnose dementia based on medical history, physical examination, laboratory tests, and formal assessments of changes in cognition, daily functioning, and behaviors [5]. Neuropsychological evaluations can be particularly useful diagnostic tools, capturing cognitive function in multiple domains, including language, attention, executive functioning, and memory abilities, but are often time-consuming and expensive. Some cognitive screening measures, such as the Mini-Mental State Examination (MMSE) and Montreal Cognitive Assessment (MoCA), are commonly used in clinical settings, such as outpatient primary care or memory disorder clinics, to assist providers with the evaluation of cognitive functioning, but are more imprecise in diagnostic accuracy.

The rise of ubiquitous sensing applications and digital health in the last decade has led to increased interest and opportunities for developing novel metrics for assessing dementia-based disorders. Existing research datasets offer opportunities to identify and validate these novel measures as potential digital biomarkers [6], but many do not integrate sensing technologies with traditional diagnostic data [e.g., demographic, neuroimaging, electronic health records (EHR), etc.]. In addition, neurocognitive research data in longitudinal studies is rarely collected at regular intervals. However, ubiquitous sensing technologies can address this limitation by enabling real-time capture of data with insights into cognition and behavior [6]. The integration of health data from each of these sources may enable the application of big data methods in diagnosing and treating dementia and facilitate improved early-stage detection [5, 7]. Of particular interest is the use of speech analysis technologies, notably psycholinguistic measurements, to measure changes in cognition and functional behavior to diagnose cognitive aging-related disorders. Discussion of speech analysis technologies has traditionally included both language and vocal speech analysis, though both language and speech are studied individually under the context of psychology [8].

Previous work demonstrated the utility of speech features in predicting the presence of dementia. Yancheva et al. [9] predicted MMSE scores using lexico-syntactic, acoustic, and semantic features from speech samples in DementiaBank, a shared database of pathological speech from healthy and dementia-inflicted participants. Members of their research team expanded upon this by successfully using linguistic and audio features extracted from the Boston Cookie Theft task [10] to distinguish between controls and participants diagnosed with “possible” or “probable” AD [11]. Similarly, Alhanai et al. [12] used speech and language features to determine the presence of cognitive impairment using data from the Framingham Heart Study (FHS). Alhanai et al. [12] reported that linguistic and audio features had similar or better predictive power than well-documented demographic risk factors of dementia (e.g., age and sex).

A recent analysis by Thomas et al. [13] extensively characterized the utility of speech features in predicting binary cognitive status (control *vs.* impaired) in a subset of the FHS Cognitive Aging Cohort, extending work by Alhanai et al. [12]. The results of the analysis notably showed an area under the AUC of 0.908 for an ElasticNet model trained on linguistic features, and improved performance (0.943) when the model incorporated demographic variables and acoustic features. Standalone acoustic features performed worse than demographic and linguistic features individually in predicting cognitive status. However, acoustic feature performance was confounded by a high signal-to-noise ratio in the recorded audio files, as noted in the analysis. This previous work agrees with existing evidence characterizing linguistic deficits and demographic covariates, notably education, as key factors in cognitive impairment [14–17].

In current clinical practice, memory disorder clinicians do not record speech data during neuropsychological examinations. With optimistic signals from prospective studies, there is a growing incentive to record and digitize cognitive examination speech data. Speech data collection is cost-effective and accessible and incurs a minimal clinical burden on clinicians and patients [18]. Speech biomarker technologies may also be incorporated into clinical decision support tools alongside existing diagnostic tools (e.g., blood tests, imaging).

Before speech biomarkers for cognitive assessment can be applied in clinical settings, much work must be done to identify and clinically validate the value of speech biomarkers, both in parallel to and in conjunction with existing diagnostic methods. To do so, we must understand how well existing methods predict cognitive status, and what further benefits speech biomarkers can provide. Speech biomarkers present an accessible, low-cost, and rapid mechanism for screening and testing compared to neuropsychological testing, which requires highly trained specialists and substantial time and cost investments. In order to characterize phenotypes and eventually biomarkers from speech features, biological data, such as blood, cerebrospinal fluid volume, and brain imaging data must be included. This work thus makes a critical contribution beyond existing work, which has focused on the connection between speech features and cognitive phenotypes, and the steps required to develop speech biomarkers. As voice recording becomes ubiquitous, the data available to strengthen these findings will grow substantially in the near future.

In this work, we provide in-depth contextual analysis of linguistic features in detecting cognitive impairment and examine how to improve their clinical viability. First, based on the optimistic performance of language in predicting cognitive status from our research team’s previous work, we pursue a deeper dive into the primary dimensions of language, including syntactic, semantic, and lexical information, to expand the feature set reported by Thomas et al. [13]. We perform an error analysis to gauge deficits of the models in predicting cognitive status in key demographic groups. Similar to our team’s previous work, we provide additional neuropsychological context of feature performance by identifying correlations between both the original and expanded linguistic feature sets and performance on neuropsychological tests (NPTs). We specifically use language-based NPTs which include the logical memory, paired association, Boston Naming, and verbal fluency tests. In comparing linguistic features to performance on NPTs, we can understand how well different dimensions of language reflect cognitive deficits in those with dementia. Finally, we discuss considerations and requirements to ensure high-quality speech data collection that can support the eventual integration of speech biomarkers into clinical practice.

## Material and methods

### Data

This study uses a subset of 141 unique participants from the FHS Cognitive Aging Cohort. The FHS is an extensive observational study capturing longitudinal, transgenerational cohort data in the US since 1948. The FHS has conducted incident studies of dementia since 1976 and first began collecting digital voice recordings of assessments in 2005. One central objective is to capture age-related cognitive changes across the entire adult lifespan [13]. The study obtains anthropomorphic, lifestyle, organ function [19], genetic [20], and other health-related phenotypic [21] data. For this study, we used a subset of these data including demographics, NPT, and speech recordings of NPTs that required a spoken response.

#### Neuropsychological test data

The NPT data includes multiple standardized tests that assess premorbid functioning (Wide Range Achievement Test [22]), attention (WAIS Digit Span), processing speed and executive functioning [Trail Making Test (TMT) A and B [23]], verbal and language abilities [Boston Naming Test (BNT) [24]; Wechsler Adult Intelligence Scale (WAIS) Similarities [25]; Controlled Oral Word Association Test, letter naming (‘FAS’), and animal naming [22]], visuoperceptual skills [Hooper Visual Organization Test (HVOT), [26]], verbal learning and memory [Weschler Memory Scale (WMS) Logical Memory; Verbal Paired Associates [25]], visual learning and memory (WMS Visual Reproduction [27]), and motor speed (Finger Tapping Test [28]). Full details about the testing protocol can be found in Au et al. [29]. It is important to note that our analysis focuses mainly on speech measures, and not all NPTs involve spoken responses; however, all available neuropsychological measures were included in analyses to provide extended cognitive context.

Alongside the domain specific NPT, FHS also included the MMSE, a widely used cognitive screening measure which includes tasks of orientation, attention, language, visuospatial skills, and memory abilities [30]. The MMSE was measured on average 2.7 ± 0.1 years from the time of the NPT. We selected the MMSE score that was closest to the time of the NPT test (e.g., average MMSE-NPT administration time difference was 1.8 ± 0.3 years). The MMSE provides a reference to measure the validity of speech analysis to screen for cognitive impairment [11]. The MMSE is scored with a cutoff for “normal” or “abnormal” relative to the participant’s education level [31]. The FHS uses this screening measure to determine whether changes in cognitive function warrant further follow-up [31] to assess for possible onset of dementia. Although some researchers have evaluated specific items on the MMSE to determine domain-specific cognitive deficits, the MMSE is not diagnostic and does not differentiate subtypes of dementia, if any [31]. Details about the MMSE general procedures can be found in Kurlowicz et al. [31].

#### Original speech analysis data

Speech recordings were obtained simply by recording the entire 60-minute NPT session during the study. Each NPT speech recording originally included both interviewer and participant audio along with electronic copies of the transcripts. As part of our previously published work [13], we utilized the transcripts provided by FHS, isolated participant dialogue, and split transcripts by cognitive assessment task. We then extracted linguistic features from the transcripts of the Logical Memory-Delayed Recall test. Linguistic features identify information contributing to the language content of the speech data. To increase the accuracy of part-of-speech (POS) tagging, punctuation was removed from the transcripts before computing tags. With the transcripts of each audio file, we extracted POS tags using the Natural Language Toolkit (NLTK) [32] and SpaCy [industrial-strength Natural Language Processing (NLP) with Python and Cython, Explosion] [33]. Additional information on the extraction of linguistic features can be found in the “Acoustic and Linguistic Feature Extraction” section of Thomas et al. [13]. We utilized a subset of data curated and described in this prior work in this study. Here, we build upon this work by extracting additional features from the same transcripts as described in section Speech preprocessing.

We also included two additional features previously extracted from prior work in Thomas et al. [13] in this analysis: pronoun-proper noun ratio and proportion of conjunctions. Previous research has shown these correlate with story recall scores and cognitive status. For example, patients diagnosed with semantic dementia produce fewer nouns, often replacing them with pronouns [34], resulting in higher pronoun-noun ratios. Similarly, patients with AD struggle to use nouns appropriately during short discourses, also leading to greater pronoun usage [35]. Furthermore, studies have shown that some forms of AD result in a decreased use of conjunctions [36]. Kempler et al. [35] suggest that speech comprehension, including pronoun and conjunction use, can be attributed to attention and working memory deficits.

#### Diagnostic classification

A FHS dementia diagnosis adjudication panel consisting of at least one neuropsychologist and one neurologist determined the participants’ cognitive status, applying the Diagnostic and Statistical Manual of Mental Disorders (DSM-IV) criteria [37] for dementia and National Institute of Neurological and Communicative Disorders and Stroke and the Alzheimer’s Disease and Related Disorders Association (NINCDS-ADRDA) [38, 39] for AD. The panel used all available information for each study participant, which may include NPT and neurology exams, FHS study and external medical records, and interviews with participants’ caregivers. Posthumously, the FHS review panel determines cognitive status at the time of death using the totality of participants’ medical and when applicable and available, nursing home records [40]. While FHS did not routinely conduct Clinical Rating Scale assessments (a key tool used to measure psychiatric symptoms), it used a comparable rating system ranging from cognitively unimpaired to severe dementia. The FHS dementia diagnosis adjudication panel assigned the following numeric values for the possible findings: 0 = control (tested as normal) or not tested, deemed normal; 0.5 = cognitively impaired, not demented; 1-1.5 = mild dementia; 2-2.5 = moderate dementia; 3 = severe dementia.

In some cases, individual participants may have participated in multiple NPT. For our analysis, each assessment for participants with multiple assessments was treated as a unique observation, due to the variable changes in data availability and cognitive status over time of participants with more than one assessment.

### Demographics

We calculated summary statistics for the demographics of age, sex, and education and then split the subjects into ‘non-present’ (control + normal cognition) and ‘present’ [mild cognitive impairment (MCI) + dementia at any level]. We performed a *t*-test to evaluate differences in age between the present and non-present groups. We also used Chi-square tests to evaluate the differences in sex and education between the present and non-present groups.

### Speech preprocessing

Part of this work was aimed to expand the linguistic feature set previously explored, and compare the two feature sets in terms of predictive power [13]. Following previous work performing similar computerized voice analyses, we extended the feature vectorizations on the Logical Memory (delayed recall) transcript from the collected audio data. We extracted new extended features characterizing syntactic, semantic, and lexical information from the provided transcripts from Logical Memory (delayed recall) audio files. A subset of featurized data processed by Thomas (Table 2 of [13]) was also used as the original linguistic feature set to compare with the following expanded linguistic feature set.

#### Expanded syntactic features

Syntactic features may reflect speech complexity. Boschi et al. [34] observed that patients afflicted with Lewy body dementia produce syntactically simpler sentences, characterizing demented speech with reduced sentence length, clauses, verb phrases, and coordinate sentences. Reduced syntactic complexity reflects impairment in discourse organization associated with right frontotemporal disease [30]. Work from Fraser et al. [11] and Orimaye et al. [41] showed notably strong predictive power in the syntactic analysis of speech from the DementiaBank clinical dataset, a highly used corpus of patients with possible or probable AD diagnoses and controls with available narrative voice samples to obtain various speech features. Both works used NLTK’s Stanford parser to extract syntactic features from participant speech such as sentence length, clause tags, and parse tree depth. In line with these works, we extract features regarding the mean length of sentences, clauses, and phrase types, and parser tree depths. Similar to Fraser et al. [11] and Orimaye et al. [41], the Stanford parser from NLTK (version 3.4) was used to extract raw counts of phrase and clause level tags specified in Penn Treebank II [42]. Additionally, we extracted parse trees from each sentence in the transcripts to calculate average and maximum tree depth.

#### Expanded semantic features

Semantic features identify information contributing to the logic and meaning of speech, forming the expressivity of language [43]. Jarrold et al. [44] characterize the speech of individuals with dementia as having smaller vocabularies for different semantic concepts than the speech of healthy people, reflecting difficulties in semantic processing and word-finding capabilities. For each transcript, individual words were categorized into semantic groups using Pennebaker et al.’s [45] Linguistic Inquiry and Word Count (LIWC) 2015 English Dictionary. In addition to the number of unique words in each category, the number of non-empty categories was calculated, capturing both the breadth and frequency of semantic categories in each participant’s speech. Compared to individuals with normal cognition, cognitively impaired individuals use fewer words to describe the same concepts, which reduces the breadth of semantic categories used in their speech. Previous works have shown LIWC semantic categorization to be a powerful identifier of cognitive impairment [44, 46].

#### Expanded lexical features

Lexical features reflect the breadth of vocabulary. When asked to describe pictures, patients with dementia provide shorter, less informative oral and written descriptions [47] compared to healthy individuals, indicating word-finding difficulties that often result in muteness in patients with dementia [48]. Orimaye et al. [41] and Fraser et al. [11] also reported strong performance of lexical features in predicting the presence of dementia We featurized the lexical information using a subset of the features in Orimaye et al. [41] (unique word count, word count, and character length). From each transcript, we calculated features regarding the number of unique words, the number of times each unique word is used, and the mean word length. For each feature, we repeated feature extraction on a version of the transcript on which lemmatization and stemming had been completed and stop words had been removed. Lemmatization and stemming effectively standardize different versions of the same words. For example, “run”, “ran” and “running” are all considered the same word after lemmatization and stemming. Stop words, such as “is”, “the”, “a”, were not directly relevant to performance on the task, and were removed to eliminate the bias of signal in our predictive models.

### Predictive models of speech data for dementia and cognitive decline

Features from the audio samples were used to train binary classification models of cognitive impairment.

#### Binary classification

We utilized statistical models to predict binary cognitive impairment status (impaired/unimpaired). We applied Logistic Regression from the scikit-learn Python package (version 0.20.3) to estimate binary cognitive status. We tested different Lasso (L1) and ridge (L2) regularization penalties as well as varying regularization parameters (λ) to prevent overfitting without a sizable loss in performance. Penalty weights of 102, 10, 1, 10-1, 10-2, 10-3, and 10-4 were tested for λ. Each model was trained utilizing leave-one-out cross validation. We then selected the highest performing regularization term and λ to use for each model when comparing their performance.

Related predictive features were grouped together into feature sets. Feature sets are as follows: NPT, MMSE, Baseline Linguistic, Syntactic, Semantic, and Lexical. The Baseline Linguistic feature set consists of the linguistic features used in our prior work [13]. Speech-based feature sets (Baseline Linguistic, Syntactic, Semantic, and Linguistic) were also paired with MMSE features and modeled to evaluate the performance of each speech-based feature set in tandem with MMSE screening. It’s important to note that NPT and MMSE are used in diagnosing dementia by FHS and therefore results have to be interpreted with caution due to issues of circular reasoning. However, the NPT feature set is still included as a comparative performance measure of traditional cognitive assessment methods. Features are normalized across individual feature sets using standard scaling from −1 to 1. The combination of MMSE and speech-based features is utilized to demonstrate the value of a data collection paradigm that may be amenable to general practice settings, prior to neuropsychological testing and diagnosis for earlier detection of cognitive impairment.

#### Model performance measurement

Classification performance is assessed in terms of several metrics. To evaluate binary classification performance, we use AUC, which characterizes predictive performance for both classes, unlike some other measures, e.g., accuracy. Additionally, we used confusion matrix summaries, including sensitivity, specificity, positive predictive value (PPV), and negative predictive value (NPV). In the case of cognitive impairment, it is optimal to maximize NPV without significantly reducing the PPV. This reduces the likelihood of a false negative result, which is important because undiagnosed dementia may result in a greater risk of hospitalization due to falling, fainting, and trauma [3]. Early diagnosis of dementia allows patients to benefit from treatments sooner and allows them and their loved ones to plan and develop support systems. However, it is also important to preserve a reasonable false positive rate (FPR), as an incorrect diagnosis of cognitive impairment or dementia can inflict emotional trauma and cause undue burdens such as medical costs or the withdrawal of their right to operate a motor vehicle. In practice, however, these risks appear to be minor in comparison to those associated with a false negative result. The decision threshold for classification was chosen to maximize the sum of sensitivity and specificity.

#### Error analysis

Previous studies have demonstrated the limitations of using MMSE to screen different demographic groups. These include impacts of age, education, sex, and race on performance on the MMSE [14, 15, 17, 49, 50]. Age and education in particular have been previously shown to have a significant influence on cognitive status [15, 16]. This may have potential confounding effects that cause incorrect predictions. To account for this in our study, we performed a Mann-Whitney *U* test to examine the effect of age and a Kruskal-Wallis test to examine the effect of education on both the male and female groups. Additionally, the false negative rate (FNR) and FPR were assessed across all demographic groups.

We also evaluate the performance of the proposed expanded linguistic model in terms of true positive rate (TPR) for sub-classifications of cognitive impairment, including MCI and dementia (mild and moderate). As we use a binary classification model, we seek to validate that the model is not solely driven by the dementia subgroup of the cognitive impairment class.

#### Neuropsychological specificity

In addition to characterizing the performance of our linguistic features in predicting cognitive status, we also sought to characterize the relationship between our linguistic features and language-dependent NPTs. Though sets of features predict cognitive status with high performance, such features should also reflect the general performance of linguistic deficits as measured by NPTs. Language-dependent NPTs characterize language function in conjunction with other dimensions of cognitive function such as verbal learning, memory, and executive function. The NPTs of interest include Logical Memory Immediate Recall, Logical Memory Delayed Recall, Logical Memory-Delayed Recognition, Paired Associate Learning Immediate Recall, Paired Associate Learning Delayed Recall, BNTcorrect of 30 item, Letter Fluency (“FAS”), and Category Naming (animals).

To assess univariate effects, we adopt the same methodology from our previous work. We applied Lasso regression on the dataset and selected features with the largest coefficients, and subsequently calculated Pearson correlations between the selected features and NPT scores. To examine multivariate effects, we perform multivariate ElasticNet regression, taking into consideration cognitive status and combinations of linguistic features to fit NPT data. Cognitive status is incorporated as a binary variable of presence *vs.* non-presence. Multivariate performance is characterized by the R2 score value. We then interpret the identified coefficients to assess individual predictor effects in the multivariate context.

## Results

### Demographics

Of the subset of 141 participants, 43 participants were excluded due to missing data for demographics, MMSE results, cognitive status, or speech recording/transcripts. This yielded a final subcohort of 98 participants for our analysis. Our study consisted of 127 observations from 98 participants. Participants without presence of cognitive impairment (*n* = 72, female = 46%, age = 72.2 ± 2.3) were significantly younger (*t*-statistic = −3.378; *P* < 0.001), more educated (Chi-square statistic=28.646; *P* < 0.001), and more commonly male sex (Chi-square statistic = 7.831; *P* = 0.005) than participants with cognitive impairment (*n* = 55, female = 51%, age = 83.4 ± 1.9). Relevant demographic information for the 127 participant observations is provided in Table 1.

### Model performance

Based on the model performance, both NPT and MMSE classify cognitive status well in isolation. NPT overall performs the best in terms of AUC (0.929) and NPV (0.938), MMSE has the highest PPV (0.900) and still shows a NPV of 0.872. Again, this is expected because the MMSE is also included in the FHS consensus diagnosis process. We provide a summary of the results of the model across each of the different sets of data in Table 2.

In comparison to cognitive examination methods, both the original and expanded linguistic features individually outperform MMSE in terms of AUC and NPV but do not outperform NPT on any metric. However, combining both sets of linguistic features with MMSE data yields an improvement over each individual feature set and has a performance approaching that of the NPT data (Original Linguistic + MMSE: AUC = 0.913, NPV = 0.900; Expanded Linguistic + MMSE: AUC = 0.915, NPV = 0.913; NPT: AUC = 0.929, NPV = 0.938).

Although the baseline linguistic feature set outperforms the newly extracted feature sets (Syntactic, Semantic, and Lexical) individually, the expanded linguistic feature set outperforms the original linguistic feature set on all key metrics of performance. The baseline and expanded linguistic features predict cognitive status with AUC of 0.882 and 0.883, respectively. However, the expanded linguistic features appear to better characterize cognitive status deficits.

### Error analysis

According to the Kruskal-Wallis test, distribution of age for the four education groups was not shown to be significantly different [H= 3.833, *P* = 0.28]. Thus, we infer that age does not have a confounding effect on education status.

**Figure 1.**
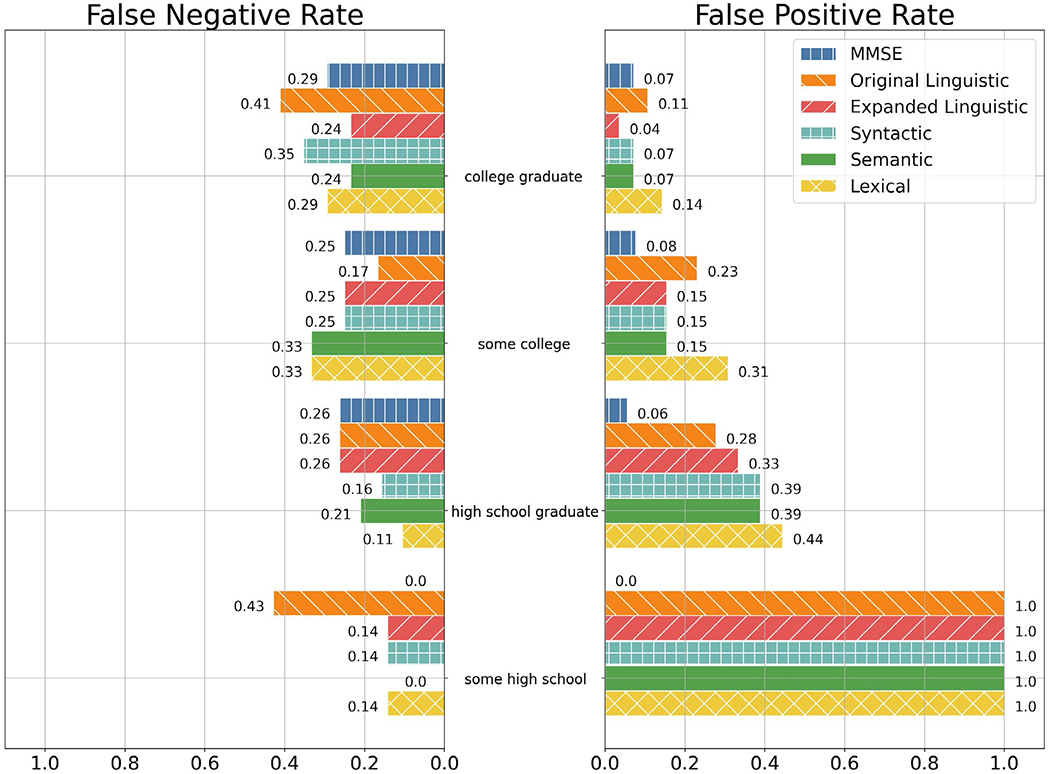
FNR and FPR of the MMSE, Baseline Linguistic, Expanded Linguistic, Expanded Syntactic, Expanded Semantic, and Expanded Lexical feature sets among education groups. Note: Lack of a bar denotes a rate of 0. Education group encoding is as follows: 0—High School, did not graduate; 1—High School; 2—Some College; 3—College Graduate. Education group 0 only had one observation of non-presence, so the only possible FPRs in this group is 1.0 and 0.0

Per Figure 1, both the baseline and expanded linguistic feature sets had improved FPRs with more educated participants. As such, the baseline and expanded linguistic feature sets were less likely to label a cognitively normal participant as impaired when the participant was more educated.

In comparison with cognitive examination methods, MMSE was less likely to give a false positive label than both the baseline and expanded linguistic feature sets for participants who have less than a college education (some high schools, high school graduates, and some colleges). However, the expanded linguistic feature set had a lower FPR than both MMSE and the baseline linguistic feature set for participants who have graduated college.

The expanded linguistic feature set had lower rates of false positives than participants with at least some college education than the baseline linguistic feature set. However, for participants with at most a high school education, the baseline linguistic feature set had an FPR lower than or identical to the expanded linguistic feature set.

Per Figure 1, MMSE was less likely to give a false negative prediction than both the baseline and expanded linguistic feature sets for participants who did not graduate high school. As such, MMSE was least likely to label a cognitively impaired participant as normal for participants who did not graduate high school. For participants with a high school diploma or greater, MMSE had an identical or greater FPR than that of the expanded linguistic feature set.

**Figure 2.**
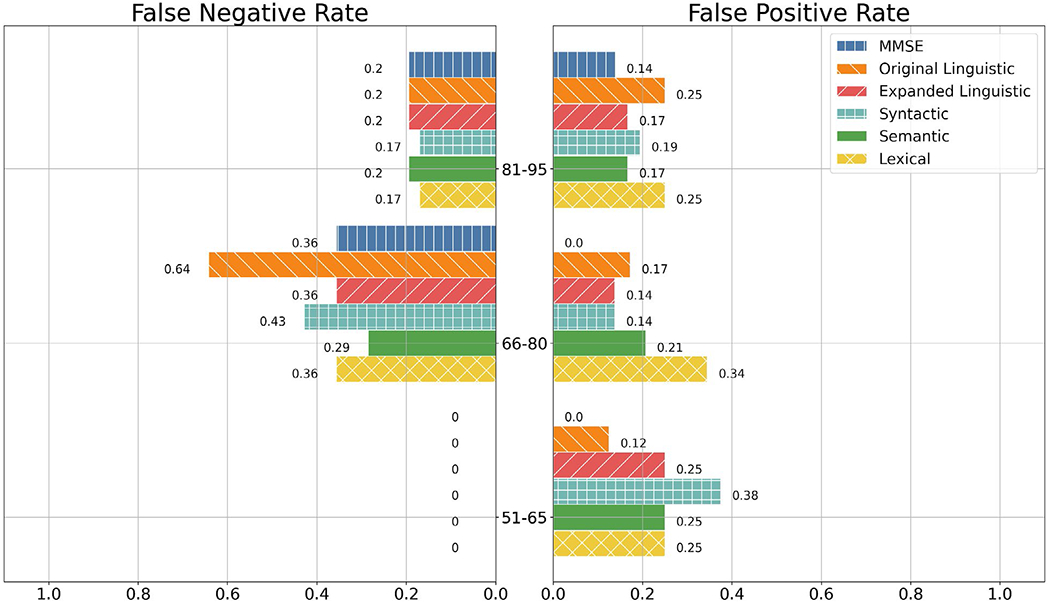
FNR and FPR of the MMSE, Linguistic, Syntactic, Semantic, and Lexical feature sets among age groups. Note: Lack of a bar denotes a proportion error of 0

Per Figure 2, MMSE had the lowest FPRs for participants less than 91 years old, but had the greatest FPR for participants 91 years old or older. In fact, MMSE gave no false positive predictions for any participant 75 years old and younger, whilst also being the sole feature set to give a false positive label for participants ages 91 and older. The baseline and expanded linguistic feature sets both tended to have lower FNRs as participants aged.

According to the Mann-Whitney test, distribution of age between males and females was not shown to be significantly different (U = 1883.5, *P* = 0.23). Thus we infer that age does not have a confounding effect on sex within our dataset.

**Figure 3.**
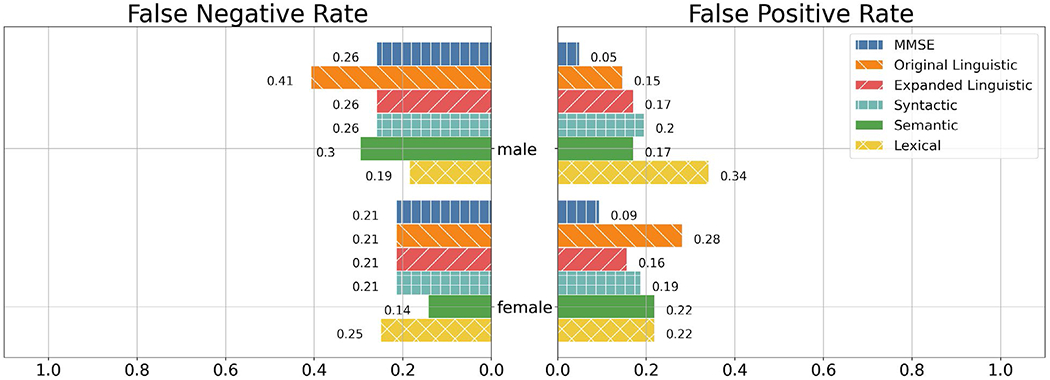
FNR and FPR of the MMSE, Linguistic, Syntactic, Semantic, and Lexical feature sets between males and females. Note: For interpretability, we only included the individual feature sets as well as the best-performing combination of feature sets

Per Figure 3, MMSE and the baseline linguistic feature set had lower FPRs on male participants than on female participants while the expanded linguistic feature set had a lower FPR on female participants than on the male participants.

Between the baseline and expanded linguistic feature sets, the baseline linguistic feature set had a lower FPR among males while the expanded linguistic feature had a lower FPR among females. However, MMSE had the lowest FPR in both groups. In both male and female participants, MMSE was the least likely to give a false positive label.

MMSE as well as the baseline and expanded linguistic feature sets all had lower FNR on female participants than they did on the male participants. All three models had identical FNR on the female demographic while the baseline linguistic feature set had greater FNR on the male demographic in relation to MMSE and the expanded linguistic feature set, which had identical FNR.

The results of subgroup analysis of the cognitive impairment groups per the expanded linguistic feature binary classification give TPR of 0.75 (9 of 12 correctly classified) for the MCI group, and 0.77 (33 of 43 correctly classified) for the dementia group. The TPR of the cognitive impairment class is 0.76 (42 of 55 correctly classified).

### Neuropsychological specificity

**Figure 4.**
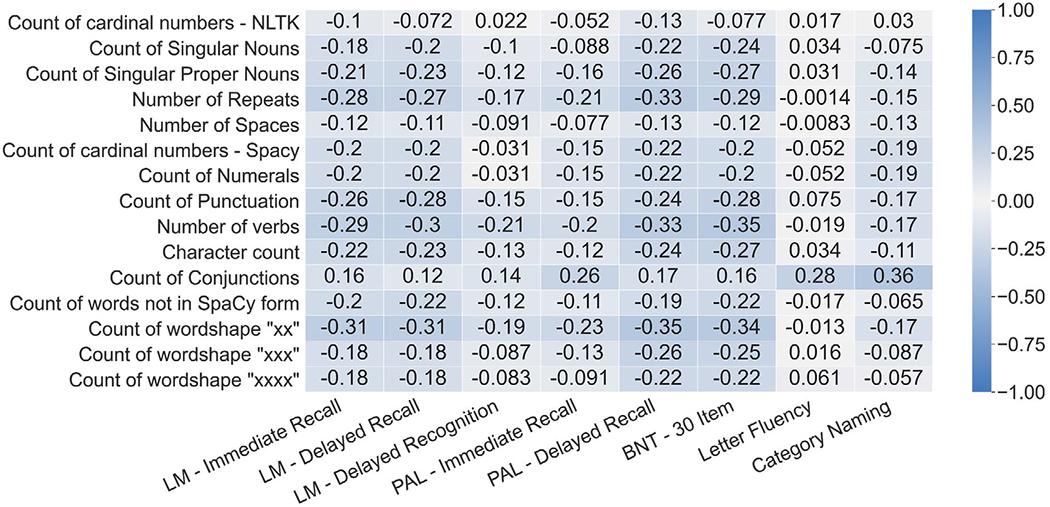
Pearson correlations between the top 15 most contributing features from the original linguistic feature set and the language-based NPTs. LM: Logical Memory; PAL: Paired Associate Learning; BNT: Boston Naming Test

**Figure 5.**
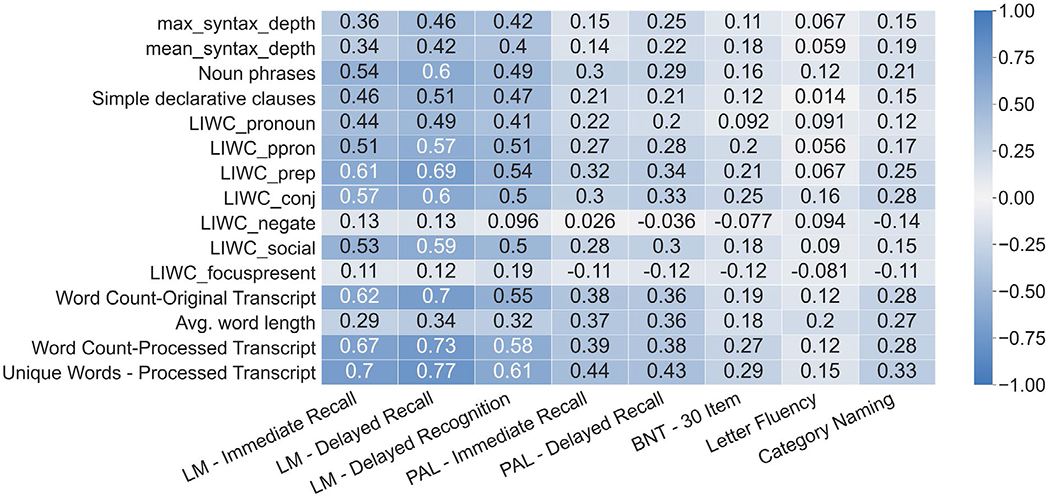
Pearson correlations between the top 15 most contributing features from the expanded linguistic feature set and the language-based NPTs

Univariate correlations between top lasso weighted features of both the original and expanded feature sets are shown in Figure 4 and Figure 5 respectively. Features in the expanded linguistic feature set are more well-correlated with the series of Logical Memory NPTs (Delayed Recalled, Immediate Recall, and Recognition) followed by the series of Paired Associate Learning NPTs (Immediate and Delayed). The expanded linguistic feature set had weak alignment with all included versions of the Boston Naming and Letter and Category Fluency NPTs. Notably, we did not analyze circumlocutions or perseverations.

Despite having low alignment with the Verbal Fluency NPTs, the expanded linguistic feature set was generally more aligned with the individual language-based NPTs than the baseline linguistic feature set. With the exception of the Boston Naming Test, the expanded linguistic feature set had correlations stronger or on par with the correlations of the baseline linguistic feature set for all language-based NPTs.

The baseline linguistic feature set most commonly had the strongest performance in predicting participant scores on language-based NPTs, as shown in Table 3. The expanded linguistic feature set had stronger performance on the Logical Memory NPTs while the baseline linguistic feature set had better predictive performance on the Paired Associate, Verbal Fluency, and 30 Item BNT NPTs.

## Discussion

Previous works lay a foundation for the measurement of cognitive decline via speech markers. Further validation of speech indicators to be used in the clinical setting is needed. By tapping into the existing clinical diagnostic framework (e.g., NPTs, MMSE), we can provide context as to which linguistics features are most well suited to predict cognitive status–through their comparison to what is currently used in clinical practice– and understand the value of speech markers.

### Considerations for clinical use

There are distinct clinical opportunities presented by the results of this analysis. The combination of the extended feature set paired with the MMSE (AUC = 0.915, NPV = 0.913) shows marginally lower performance in predicting the presence of cognitive impairment than the NPT (AUC = 0.929, NPV = 0.938). This suggests potential value for incorporating speech recordings for the general practice settings. Given the improved performance with the additional linguistic features, the decomposition of language into syntactic, semantic, and lexical behavior may be a better representation of linguistic behavior in cognitive decline. The incorporation of speech technology into general practice may allow for earlier detection of cognitive impairment, and provide richer information for clinicians and researchers alike to understand disease progression and to improve treatments for dementia patients. However, the generalizability of these results must be verified on larger representative datasets before serious consideration in the clinical setting. Although the performance of language features here did not exceed that of traditional NPT, this was expected and does not diminish from the primary value of using voice features coming from the lower cost, immediate availability, ease of access from a patients’ home, standardization, and removal of the provider availability bottleneck. For these reasons, voice features do not necessarily need to outperform NPT to provide significant clinical value given the factors listed above. Where voice may be most beneficial is in clinical scenarios where NPT and screening tools are difficult to access or limited by time availability. We often see very long wait times for NPT and digital tools could also be used to triage and further screen patients before sitting down for full NPT in order to help reduce the burden on neuropsychologists.

Speech analysis can also begin by filling gaps in current practice that are difficult to address with screening tools such as MMSE and full NPT. In populations challenging to reach with traditional measures, such as remote and minority populations, voice analysis could be a means of providing access to testing. Voice analysis also provides the ability to screen and test far more individuals per provider by removing the bottleneck of provider time required. During the global pandemic we face, voice analysis also presents an avenue for remote testing, alleviating the burden of limited in person access. Our voice analysis here outperformed MMSE in those with higher education; this may be another gap where voice analysis could provide better results than traditional screening tools.

Before novel markers can be considered for clinical use, they must demonstrate effective predictive value that outweighs the cost of data collection. As mentioned, collection of speech recordings is fairly cost effective. Availability of a single microphone or appropriate recording device is sufficient for data collection for many exams. However, there may be additional cost factors. In order to acquire linguistic information, transcription is required. Transcription costs can range from as low as $0.50 an hour (automated speech recognition (ASR) Speech-to-Text) to up to ~ $30 an hour (Manual Transcription).

### Statistical modeling

The demographic reporting of participants in Table 1 demonstrates relatively even distribution of participant age, sex, and education level across the cognitive categories. The resulting model performance shown in Table 2 demonstrates high performance for NPT features alone, as expected, given the extensive testing and long validation track record. The sensitivity of models trained on NPT features in detecting cognitive impairment was higher compared to that of other models, which is not surprising given the thoroughness of NPT. However, the specificity of NPT was lower compared to other models. This was also unsurprising, considering that NPT assesses performance on specific cognitive tasks rather than daily functioning. Given the heterogeneity of cognition, assessments conducted periodically fail to capture the full range of cognitive capabilities that are underlying daily functional behaviors. For this reason, NPT may provide an erroneous result. For example, if a participant did not sleep well the previous night, their NPT scores may be affected even if their cognition is intact. This is reflected in the PPV for NPT as well. Speech based features may also be sensitive to similar effects including sleeplessness, fatigue, and time-of-day at the time of recording from which features are calculated.

The better specificity of MMSE compared to NPT alone was expected given the MMSE is a screening tool specifically designed to detect a general state of cognitive impairment and dementia. NPT, on the other hand, is a comprehensive set of cognitive assessments that measures domain-specific skills. The brevity of the MMSE improves accessibility at the cost of sensitivity. This is characterized by the ceiling effect of the MMSE on characterizing performance around the normal cognitive range [51]. It is important to reiterate that the performance of MMSE and NPTs should be interpreted with caution, as both are designed and used as tools to assess cognitive status with levels of sensitivity and specificity in mind. The use of MMSE and NPT use in predicting cognitive status is circular in nature, as both are in part used for diagnosis.

The expanded linguistic features perform marginally better than the baseline linguistic features on both AUC and NPV performance. This may be due to the inclusion of features representative of the structure and meaning of language which is more representative of language deficits in dementia. Specifically, the baseline feature set focused on the presence and frequency of parts of speech, whereas the expanded feature set includes features related to semantic clusterings, which demonstrate the richness of vocabulary. The expanded features also include measures of sentence length, structure, and word frequency, which mirror the complexity of language, revealing loss of diversity of vocabulary due to cognitive decline.

Similarly, adding MMSE provided a modest improvement in classification accuracy. When inspecting the performance of linguistic features as separately grouped by semantic, lexical, and syntactic features, the semantic feature set appears to perform best overall, with better sensitivity. This is also as expected, given dementia is expected to affect the ability to use word meaning accurately. Syntactic and lexical features provided high performance individually and each set of features demonstrated small but consistent improvements with the addition of MMSE.

NPT was not combined with linguistic features or other features due to the potential circularity issues between their use in diagnosis and their use in analysis. Since the diagnoses are based on clinical material available including NPT, using NPT as a separate feature would be inherently biased. Despite the potential circularity issues, MMSE was included with the expanded and original linguistic features to show the potential value of utilizing speech analysis in a general practice setting. Neuropsychological examinations are not conducted during general visits, thus showing the effective performance of pairing speech recording data with MMSE could show the value of conducting speech recordings during general visits, and could enable earlier detection of cognitive decline.

We also looked at the role of demographics in our error analysis. Specifically, higher levels of education tended to cause higher FNR s, while less education tended to cause higher FPRs. This was expected given the known influence of education on cognitive decline later in life. More educated participants, even if experiencing neurodegeneration, are likely to maintain function longer than those with less education. This is consistent with previous findings in literature and the understanding that education is a protective factor for the development of cognitive impairment [52]. Those with higher education likely are able to maintain higher performance on standard tests even while neurodegenerative burden builds in the brain. Those with less education likely struggle more with cognitive testing even without any neurodegenerative burden, due to cognitive tests inherently being reflective, to some degree, on past education. This makes cognitive testing somewhat biased with higher sensitivity in those with less education. This reflects the need to consider how cognitive tests would need to be different to accommodate those with lower education.

Age also displayed a significant impact on the error rates with lower ages causing more false negatives. Given several cognitive domains naturally decline modestly with age, this likely leads to missing cases of cognitive impairment at younger ages while symptoms are mild. Interestingly, increasing age did not appear to significantly increase the FPR but did lower the FNR e. This may be due to an increasing proportion of impaired individuals at older ages, where we know linguistic features are sensitive to detecting language changes. Sex also did not appear to play a significant role in the error rates.

It is important to recognize that the subset of participants in the study is not a cross-sectional random sample of the aging population. The subset of participants in the study is an older population and contains few individuals who have not completed high school education. Analysis of predictive models with larger cohort groups may show more realistic FPRs and FNR s for those demographic subgroups.

Subgroup analysis of the cognitive impairment class in the binary classification model fitted on our expanded linguistic features shows marginal difference between the MCI and dementia subgroups, suggesting that the classification performance of the cognitive impairment class is driven by neither the MCI nor dementia group. The effect of worsening dementia on language capacities may still drive performance of models, thus metrics such as TPR of subgroups of cognitive impairment should be studied in larger datasets, to establish generalizability of models driven by linguistic features to effectively predict cognitive impairment in subgroups.

### Neuropsychological specificity

As identified in a previous review on language deficits in neurodegenerative disorders [34], several components of language including lexical, semantic, and syntactic processing show impairment in these disorders. The extraction of the original set of linguistic features relied primarily on POS tagging, general lexical content, and structure features to characterize language, capturing lexico-syntactic information in language. Extending the dataset with features tailored to capture lexical, semantic, and syntactic information showed improvement in univariate correlations with respect to Logical Memory Recall and Paired Associate Learning tasks. Improvement is observed in the multivariate regression with respect to the Logical Memory Recall tasks, but not the Paired Associate Learning tasks (see Figure 4, 5 and Table 3). There is no empirical gain in performance for the Boston Naming Test tasks and Verbal Fluency Tasks with expanded features. This could be influenced by the nature of the tasks. BNT relies on accurate naming of presented visual stimuli, and the features used here likely are not sensitive to changes in cognition causing stimuli to be named incorrectly. The category naming task requires individuals to verbalize words of particular conceptual categories, such as animal or vegetable naming. The letter fluency task [Controlled Word Association Test (COWAT)] prompts individuals with phonemic categories, such as naming words that start with “F”, “A”, or “S”. The linguistic features extracted for this analysis do not take into consideration the restriction of linguistic content of the verbal fluency task, and are better applied to general tasks which elicit more naturalistic responses. Further analysis of the verbal fluency task is required, through the lens of existing methods such as semantic network analysis [53]. Audio features and other contextual audio data such as prosody, pauses, filler vocalizations (e.g., “uhh”) also provide valuable information which was not included in this analysis.

Though predictive power of the extended linguistic featureset (AUC = 0.883, NPV = 0.869) does not improve significantly over the predictive power of the original linguistic featureset (AUC = 0.882, NPV = 0. 839), the improvement in neurological specificity of the expanded features in some language-based NPTs suggests a better characterization of language-based phenotypes of cognitive impairment. Further analysis and verification with neuroimaging biomarkers will allow us to better assess the neurological validity of language features. The key is to balance the validation using NPT, which is a proxy for cognitive function, with real world outcomes such as driving ability, falls, medication management, etc. Speech features have the potential to characterize real world function, which is the ultimate goal of any cognitive test. It will be important in future work to use these outcomes in the validation of digital biomarkers in addition to proxies such as NPT. Notably, NPT alone is able to predict cognitive status with very few false negatives. Depending on the functional domain which may be impaired, speech features will have variable performance depending on their focus (e.g., syntax, semantics, lexical). It will be useful to build predictive models of functional decline using targeted features.

### Limitations

The current work has a number of limitations in common with our prior work [13]. Audio quality was a limitation in our study: because many of the legacy recordings from the FHS cohort are of insufficient quality to achieve high accuracy transcription using automated services, recordings must be manually transcribed, which is prohibitively expensive and time consuming. As a result, our sample size was significantly limited, representing a small fraction of the entire dataset. The issue of quality from automated transcription is one that persists even for prospectively ascertained recordings, and is an overall limitation in the field of speech analysis at this time.

It is well documented that the prevalence of dementia as well as the performance of MMSE as a screening tool for dementia varies by ethnicity [54, 55]. FHS participants are largely Caucasian and thus results may not be generalizable to the more ethnically and racially diverse U.S. population.

In our cohort, there were significant and variable lengths of time between MMSE and NPT, making direct comparison difficult due to possible changes in participant cognitive function. Participants diagnosed with severe dementia lacked MMSE data and were consequently removed, limiting the range of comparison.

As in our previous work, we used a binary approach, rather than a multiclass approach, to characterize the power of speech in predicting cognitive status. The binary approach addresses the contemporary limitation of data availability for this study. As outlined in Thomas et al. [13], this approach may be flawed because a participant who is mildly cognitively impaired may still have the mental capacity to “speak around” their impairment, and speak in a different manner than both cognitively normal and demented participants. However, we demonstrated that the binary approach still shows potential power to accurately predict binary cognitive status. Additionally, although cognitive impairment is sometimes a precursor to dementia, this is not always the case. It is well documented that MCI is an unstable diagnosis with significant percentages of participants reverting back to normal [56]. Therefore, it may not be appropriate to include participants with diagnosis of MCI in the same class as dementia. Orimaye et al. [41] suggest that creating a third class for cognitively impaired, but not demented, participants may be a stronger approach. However, due to the sample size limitation, adjusting our approach accordingly was not feasible for this work.

Similarly, the cognitively normal group may not have been appropriately constructed, as it contains participants presumed to be normal but not all were reviewed by the diagnostic panel to confirm this assumption. Another limitation is that the transcripts we utilized for featurization may not be fully representative of a persons’ natural speech. Because we utilized transcripts from the Logical Memory (Delayed Recall) task, the performance of the Expanded and baseline linguistic feature sets may be specific to the utilized NPT transcript. It is possible the performance of speech analysis in predicting cognitive impairment may deteriorate when performing speech analysis on regular natural speech. Thomas et al. [13] give details on this limitation. A use case of NPT-specific speech analysis in conjunction with NPTs may be considered. However, participant NPTs are used in determining dementia diagnosis by FHS and are therefore a confounding variable in the diagnosis of dementia and cognitive impairment.

An important consideration for introducing novel methods like speech analysis is the repeatability of such methods. Repeatability studies have been utilized to establish the validity of our existing measurement paradigms such as traditional NPTs and MMSE. Due to the limited sample size and limited re-testing, we did not pursue repeatability testing directly in this dataset. One benefit of the study design we pursued to train the model is the use of data from a repeatable test with a standardizable prompt. These constraints the variability of linguistic responses and may enforce repeatability.

A recent study on repeatability of speech and language based features in dementia diagnosis encapsulates similar dimensions of analysis on language features (syntactic, lexical, and semantic) and demonstrates that there are features that do repeat well, evidenced by high intraclass correlations within subjects [57]. Repeatability studies are still scarce in automated speech and language analyses, and different analyses often identify different feature sets of interest. However, with scaled up-sample sizes and more re-testing, we can pursue repeatability analysis of features.

Though omitted from the study, acoustic analysis of speech has a key advantage over linguistic analysis in its cost. As data sample size scales up, it will be important to further explore the predictive power of acoustic information and how the combination of acoustic and linguistic information as speech biomarkers can show improvement in predictive value. A recent analysis on acoustic information shows such promising predictive value of acoustic information on a larger subset (4,849 participants) of the FHS Cognitive Aging Cohort [59]. When considering speech biomarkers, the cost of transcription should be factored into the cost-benefit analysis as it pertains to clinical value.

### Future work

Future work toward establishing digital speech biomarkers for cognitive decline requires incorporation of biological and anatomical data. If normalized data can be obtained, we would analyze speech data alongside brain magnetic resonance imaging (MRI) data to search for patterns and to validate any speech-anatomy correlations. Most interesting perhaps would be an analysis on the larger FHS cohort with inclusion of imaging biomarkers. The FHS dataset represents a major avenue to answer questions regarding the existence and utility of speech biomarkers. Other works have documented the association between MRI, blood data, and dementia [58]. Further, and perhaps most importantly, it is critical to expand analysis to include ethnically and culturally diverse populations to make every effort to eliminate inequity in future biomarker development. We would also like to explore the utility of speech features in detecting specific dementia subtypes which may provide avenues to capture phenotypic differences detectable through voice features.

In conclusion, in this study, we built on the previous linguistic analysis performed by Thomas et al. [13], by incorporating additional context including neurocognitive examination data and additional lexical, semantic, and syntactic features. By identifying key dimensions of speech affected by dementia based language deficits, we were able to show improved performance in predicting cognitive status over the features in the previous analysis. The expanded linguistic feature set also showed stronger correlation with both Logical Memory Recall and Paired Associative Learning tasks, showing better specificity in characterizing cognitive deficits.

Our analysis focuses on a small subset (~ 2%) of the entire FHS Cognitive Aging Cohort which consists of 9,000+ participants. As the available corpus of cognitive aging data expands, normative evaluation of novel markers becomes more important. Our inclusion of neurocognitive examination data into cognitive status prediction provides the context by which the predictive power of such novel markers can be evaluated. Future analysis will include larger datasets and naturalistic speech to explore the utility of speech as a proxy for formal NPT and detection of functional impairment.

This work demonstrated the potential of speech features to help close gaps in neuropsychological assessment and diagnosis. This includes in areas where MMSE, or other screening tools, fall short, such as in those with lower education or less access to healthcare providers. In these scenarios, speech may be a supplement or even substitute for traditional screening tools.

## Figures and Tables

**Table 1.** Distribution of samples across different demographic groups

Demographic	Total	Cognitively normal			Cognitively impaired				*P*-value
		Total	Control (Tested as normal)	Normal cognition (Not tested, deemed normal)	Total	MCI	Mild dementia	Moderate dementia	
Size, *n*	127	72	60	13	55	12	26	17	NA
Education group (Mode)	College graduate (35%)	College graduate (38%)	College graduate (47%)	Some college (61%)	High school graduate (35%)	College graduate (58%)	High school graduate (46%)	High school graduate (35%)	< 0.001
Sex (M/F)	67/60	41/32	36/24	5/8	27/28	8/4	11/15	8/9	0.005
Age (SD)	80.5 ± 1.6	72.2 ± 2.3	77.8 ± 2.6	80.0 ± 4.9	83.4 ± 1.9	81.1 ± 5.3	83.8 ± 2.5	84.6 ± 3.5	< 0.001

SD: standard deviation

**Table 2.** Model performances for classification of presence of any cognitive impairment among different feature sets (MCI and dementia are grouped together). Thresholds balance out the TPR and FPR

Feature set	Penalty	λ	AUC	Specificity	Sensitivity	PPV	NPV	Number of Features
NPT	L1	10	0.929	0.836	0.927	0.81	0.938	23
MMSE	L1	1	0.870	0.932	0.818	0.900	0.872	18
Baseline Linguistic	L2	10	0.882	0.726	0.855	0.701	0.869	374
Baseline Linguistic + MMSE	L1	1	0.913	0.863	0.873	0.828	0.900	392
Expanded Linguistic	L2	100	0.883	0.767	0.873	0.738	0.889	163
Expanded Linguistic + MMSE	L1	10	0.915	0.863	0.891	0.831	0.913	181
Expanded Syntactic	L2	10	0.821	0.808	0.764	0.75	0.819	31
Expanded Semantic	L2	100	0.870	0.795	0.836	0.754	0.866	126
Expanded Lexical	L1	10	0.844	1.000	0.618	1.000	0.777	6

**Table 3.** R2 scores of the feature sets in predicting participant performance on language-based NPTs

Exam	Feature set				
	Original linguistic	Expanded linguistic	Syntactic	Semantic	Lexical
Logical Memory Immediate Recall	0.468	0.579	0.479	0.556	0.552
Logical Memory Delayed Recall	0.489	0.66	0.543	0.616	0.634
Logical Memory Recognition	0.167	0.296	0.221	0.266	0.282
Paired Associate Learning Immediate Recall	0.318	0.309	0.264	0.281	0.308
Paired Associate Learning Delayed Recall	0.282	0.25	0.232	0.24	0.25
BNT correct without cues for 30 item	0.181	0.144	0.096	0.115	0.094
Letter Fluency	0.413	0.305	0.231	0.284	0.145
Category Naming	0.37	0.324	0.328	0.306	0.331

## Data Availability

Given that the text transcripts, demographic, and neuropsychological test data contain personal information, the dataset used in the current study is not publicly available. However, the scripts and tools are available upon request.

## Abbreviations

AD: Alzheimer’s disease
AUC: area under the receiver-operator curve
BNT: Boston Naming Test
FHS: Framingham Heart Study
FNR: false negative rate
FPR: false positive rate
MCI: mild cognitive impairment
MMSE: Mini-Mental State Examination
NLTK: Natural Language Toolkit
NPTs: neuropsychological tests
NPV: negative predictive value
POS: part-of-speech
PPV: positive predictive value
SD: standard deviation
TPR: true positive rate

## References

[1] Dementia. WHO; c2021 [cited 2020 Jun 17]. Available from: https://www.who.int/news-room/fact-sheets/detail/dementia

[2] Arrighi HM, Neumann PJ, Lieberburg IM, Townsend RJ. Lethality of Alzheimer disease and its impact on nursing home placement. Alzheimer Dis Assoc Disord. 2010;24:90–5. 19568155 10.1097/WAD.0b013e31819fe7d1

[3] Alzheimer’s facts and figures report. Alzheimer’s Association; c2021 [cited 2020 May 26]. Available from: https://www.alz.org/alzheimers-dementia/facts-figures

[4] de Vugt ME, Verhey FRJ. The impact of early dementia diagnosis and intervention on informal caregivers. Prog Neurobiol. 2013;110:54–62. 23689068 10.1016/j.pneurobio.2013.04.005

[5] Research C for DE and. Alzheimer’s disease: developing drugs for treatment guidance forindusty. US Food Drug Adm; [cited 2020 Aug 31]. Available from: https://www.fda.gov/regulatory-information/search-fda-guidance-documents/alzheimers-disease-developing-drugs-treatment-guidance-industy

[6] Zhang R, Simon G, Yu F. Advancing Alzheimer’s research: a review of big data promises. Int J Med Inform. 2017;106:48–56. 28870383 10.1016/j.ijmedinf.2017.07.002 PMC5590222

[7] U.S. Department of Health and Human Services. National plan to address Alzheimer’s disease: 2018 Update [Internet]. 2018. [cited 2020 Aug 31]. Available from: https://aspe.hhs.gov/system/files/pdf/259581/NatPlan2018.pdf

[8] Robin J, Harrison JE, Kaufman LD, Rudzicz F, Simpson W, Yancheva M. Evaluation of speech-based digital biomarkers: review and recommendations. Digit Biomark. 2020;4:99–108. 33251474 10.1159/000510820 PMC7670321

[9] Yancheva M, Fraser K, Rudzicz F. Using linguistic features longitudinally to predict clinical scores for Alzheimer’s disease and related dementias. Proceedings of SLPAT 2015: 6th Workshop on Speech and Language Processing for Assistive Technologies; 2015 Sep 11; Dresden, Germany. ACL Anthol; 2015.

[10] Mack WJ, Freed DM, Williams BW, Henderson VW. Boston naming test: shortened versions for use in Alzheimer’s disease. J Gerontol. 1992;47:P154–8. 1573197 10.1093/geronj/47.3.p154

[11] Fraser KC, Meltzer JA, Rudzicz F. Linguistic features identify Alzheimer’s disease in narrative speech. J Alzheimers Dis. 2016;49:407–22. 26484921 10.3233/JAD-150520

[12] Alhanai T, Au R, Glass J. Spoken language biomarkers for detecting cognitive impairment. arXiv:1710.07551v1 [Preprint]. 2017 [cited 2020 June 15]:[8 p.]. Available from https://arxiv.org/abs/1710.07551.

[13] Thomas JA, Burkhardt HA, Chaudhry S, Ngo AD, Sharma S, Zhang L, Assessing the utility of language and voice biomarkers to predict cognitive impairment in the Framingham Heart Study cognitive aging cohort data. J Alzheimers Dis. 2020;76:905–22. 32568190 10.3233/JAD-190783

[14] Uhlmann RF, Larson EB. Effect of education on the Mini-Mental State Examination as a screening test for dementia. J Am Geriatr Soc. 1991;39:876–80. 1885862 10.1111/j.1532-5415.1991.tb04454.x

[15] Matthews F, Marioni R, Brayne C; Medical Research Council Cognitive Function and Ageing Study. Examining the influence of gender, education, social class and birth cohort on MMSE tracking over time: a population-based prospective cohort study. BMC Geriatr. 2012;12:45. 22889350 10.1186/1471-2318-12-45 PMC3542122

[16] Orrell M, Sahakian B. Education and dementia. BMJ. 1995;310:951–2. 7728017 10.1136/bmj.310.6985.951 PMC2549351

[17] Buckwalter JG, Sobel E, Dunn ME, Diz MM, Henderson VW. Gender differences on a brief measure of cognitive functioning in Alzheimer’s disease. Arch Neurol. 1993;50:757–60. 8323481 10.1001/archneur.1993.00540070069018

[18] Coravos A, Goldsack JC, Karlin DR, Nebeker C, Perakslis E, Zimmerman N, Digital medicine: a primer on measurement. Digit Biomark. 2019;3:31–71. 32095767 10.1159/000500413 PMC7015383

[19] Andersson C, Johnson AD, Benjamin EJ, Levy D, Vasan RS. 70-year legacy of the Framingham Heart Study. Nat Rev Cardiol. 2019;16:687–98. 31065045 10.1038/s41569-019-0202-5

[20] Framingham Heart Study-Cohort (FHS-Cohort). BioLINCC; [cited 2020 June 15]. Available from https://biolincc.nhlbi.nih.gov/studies/framcohort/

[21] Framingham Cohort. dbGaP Study Accession: phs000007.v31.p12 [dataset]. 3 30, 2020 [cited 2020 June 15]. Available from: https://www.ncbi.nlm.nih.gov/projects/gap/cgibin/study.cgi?study_id=phs000007.v31.p12

[22] Wide range achievement test | fourth edition. Pearson; c1996-2021 [cited 2020 June 16]. Available from https://www.pearsonassessments.com/store/usassessments/en/Store/Professional-Assessments/Academic-Learning/Wide-Range-Achievement-Test-%7C-Fourth-Edition/p/100001722.html

[23] Armitage SG. An analysis of certain psychological tests used for the evaluation of brain injury. Psychol Monogr. 1946;60:i–48.

[24] Weintraub S Boston naming test - second edition. Pearson Clinical Australia & New Zealand. [cited 2020 June 16]. Available from https://www.pearsonclinical.com.au/products/view/525

[25] Wechsler D A standardized memory scale for clinical use. J Psychol. 1945;19:87–95.

[26] Hooper E (VOT™) Hooper visual organization test™. WPS; c2021 [cited 2020 June 16]. Available from https://www.wpspublish.com/vot-hooper-visual-organization-test

[27] Downer B, Fardo DW, Schmitt FA. A summary score for the Framingham Heart Study neuropsychological battery. J Aging Health. 2015;27:1199–222. 25804903 10.1177/0898264315577590 PMC4603385

[28] Shimoyama I, Ninchoji T, Uemura K. The finger-tapping test. A quantitative analysis. Arch Neurol 1990;47:681–4. 2346396 10.1001/archneur.1990.00530060095025

[29] Brain Health Research Lab/ neuropsychology group at the Framingham Heart Study [Internet]. Boston: Boston University School of Medicine. [cited 2020 June 16]. Available from https://www.bumc.bu.edu/anatneuro/research/brain-health-research-lab-neuropsychology-group-at-the-framingham-heart-study/

[30] Ash S, Moore P, Antani S, McCawley G, Work M, Grossman M. Trying to tell a tale: discourse impairments in progressive aphasia and frontotemporal dementia. Neurology. 2006;66:1405–13. 16682675 10.1212/01.wnl.0000210435.72614.38

[31] Kurlowicz L, Wallace M. The mini-mental state examination (MMSE). J Gerontol Nurs. 1999;25:8–9. 10.3928/0098-9134-19990501-08 10578759

[32] Lobur M, Romanyuk A, Romanyshyn M. Using NLTK for educational and scientific purposes. 2011 11th Int. Conf. Exp. Des. Appl. CAD Syst. Microelectron. CADSM, 2011, p. 426–8.

[33] Honnibal M, Montani I. SpaCy: industrial-strength natural language processing (NLP) with Python and Cython. Explosion; 2019.

[34] Boschi V, Catricalà E, Consonni M, Chesi C, Moro A, Cappa SF. Connected speech in neurodegenerative language disorders: a review. Front Psychol. 2017;8. 10.3389/fpsyg.2017.00269 PMC5337522 28321196

[35] Kempler D, Goral M. Language and dementia: neuropsychological aspects. Annu Rev Appl Linguist. 2008;28:73–90. 21072322 10.1017/S0267190508080045 PMC2976058

[36] A speech recognition-based solution for the automatic detection of mild cognitive impairment from spontaneous speech. Curr Alzheimer Res; c2021 (cited 2020 February 6). Available from http://www.eurekaselect.com/157444/article 10.2174/1567205014666171121114930 PMC5815089 29165085

[37] Association AP. Diagnostic and statistical manual of mental disorders (DSM-5®). American Psychiatric Assoc; 2013. 10.1590/s2317-17822013000200017 24413388

[38] Bachman DL, Wolf PA, Linn R, Knoefel JE, Cobb J, Belanger A, Prevalence of dementia and probable senile dementia of the Alzheimer type in the Framingham Study. Neurology 1992;42:115–9. 1734291 10.1212/wnl.42.1.115

[39] McKhann G, Drachman D, Folstein M, Katzman R, Price D, Stadlan EM. Clinical diagnosis of Alzheimer’s disease. Neurology 1984;34:939. 6610841 10.1212/wnl.34.7.939

[40] Satizabal CL, Beiser AS, Chouraki V, Chêne G, Dufouil C, Seshadri S. Incidence of dementia over three decades in the Framingham Heart Study. N Engl J Med 2016;374:523–32. 26863354 10.1056/NEJMoa1504327 PMC4943081

[41] Orimaye SO, Wong JS-M, Golden KJ. Learning predictive linguistic features for Alzheimer’s disease and related dementias using verbal utterances. Proc. Workshop Comput. Linguist. Clin. Psychol. Linguist. Signal Clin. Real., Baltimore, Maryland, USA: Association for Computational Linguistics; 2014, p. 78–87.

[42] Taylor A, Marcus M, Santorini B. The Penn Treebank: an overview. In: Abeillé A, editor. Treebanks, vol. 20, Dordrecht: Springer Netherlands; 2003, pp. 5–22.

[43] Hugo J, Ganguli M. Dementia and cognitive impairment: epidemiology, diagnosis, and treatment. Clin Geriatr Med. 2014;30:421–42. 25037289 10.1016/j.cger.2014.04.001 PMC4104432

[44] Jarrold W, Peintner B, Wilkins D, Vergryi D, Richey C, Gorno-Tempini ML, Aided diagnosis of dementia type through computer-based analysis of spontaneous speech. Proc. Workshop Comput. Linguist. Clin. Psychol. Linguist. Signal Clin. Real., Baltimore, Maryland, USA: Association for Computational Linguistics; 2014, pp. 27–37.

[45] Tausczik YR, Pennebaker JW. The psychological meaning of words: LIWC and computerized text analysis methods. J Lang Soc Psychol. 2010;29:24–54.

[46] Weiner J, Schultz T. Automatic screening for transition into dementia using speech. Speech Commun. 13th ITG-Symp. 2018, pp. 1–5.

[47] Croisile B, Ska B, Brabant MJ, Duchene A, Lepage Y, Aimard G, Comparative study of oral and written picture description in patients with Alzheimer’s disease. Brain Lang. 1996;53:1–19. 8722896 10.1006/brln.1996.0033

[48] Goedert M, Ghetti B, Spillantini MG. Frontotemporal dementia: implications for understanding Alzheimer disease. Cold Spring Harb Perspect Med. 2012;2:a006254. 22355793 10.1101/cshperspect.a006254 PMC3281593

[49] Dai T, Davey A, Woodard JL, Miller LS, Gondo Y, Kim S-H, Sources of variation on the Mini-Mental State Examination in a population-based sample of centenarians. J Am Geriatr Soc. 2013;61:1369–76. 23889552 10.1111/jgs.12370 PMC3743957

[50] Tangalos EG, Smith GE, Ivnik RJ, Petersen RC, Kokmen E, Kurland LT, The Mini-Mental State Examination in general medical practice: clinical utility and acceptance. Mayo Clin Proc. 1996;71:829–37. 8790257 10.4065/71.9.829

[51] Trzepacz PT, Hochstetler H, Wang S, Walker B, Saykin AJ. Relationship between the Montreal Cognitive Assessment and Mini-mental State Examination for assessment of mild cognitive impairment in older adults. BMC Geriatr. 2015;15. 10.1186/s12877-015-0103-3 PMC4562190 26346644

[52] Lesuis SL, Hoeijmakers L, Korosi A, de Rooij SR, Swaab DF, Kessels HW, Vulnerability and resilience to Alzheimer’s disease: early life conditions modulate neuropathology and determine cognitive reserve. Alzheimers Res Ther. 2018;10:95. 30227888 10.1186/s13195-018-0422-7 PMC6145191

[53] Zemla JC, Austerweil JL. Analyzing Knowledge Retrieval Impairments Associated with Alzheimer’s disease using network analyses. Complexity. 2019;2019:e4203158. 10.1155/2019/4203158 PMC6656530 31341377

[54] Harwood DG, Ownby RL. Ethnicity and dementia. Curr Psychiatry Rep. 2000;2:40–5. 11122930 10.1007/s11920-000-0040-4

[55] Leveille SG, Guralnik JM, Ferrucci L, Corti MC, Kasper J, Fried LP. Black/white differences in the relationship between MMSE scores and disability: the women’s health and aging study. J Gerontol Ser B. 1998;53B:P201–8. 10.1093/geronb/53b.3.p201 9602835

[56] Overton M, Pihlsgård M, Elmståhl S. Diagnostic stability of mild cognitive impairment, and predictors of reversion to normal cognitive functioning. Dement Geriatr Cogn Disord. 2019;48:317–29. 32224608 10.1159/000506255

[57] Stegmann GM, Hahn S, Liss J, Shefner J, Rutkove SB, Kawabata K, Repeatability of commonly used speech and language features for clinical applications. Digit Biomark. 2020;4:109–22. 33442573 10.1159/000511671 PMC7772887

[58] Liu CK, Miller BL, Cummings JL, Mehringer CM, Goldberg MA, Howng SL, A quantitative MRI study of vascular dementia. Neurology. 1992;42:138. 1734295 10.1212/wnl.42.1.138

[59] Lin H, Karjadi C, Ang TFA, Prajakta J, McManus C, Alhanai TW, Identification of digital voice biomarkers for cognitive health. Explor Med. 2020;1:406–17. 33665648 10.37349/emed.2020.00028 PMC7929495

